# Supplementary material for: Allosteric regulation accompanied by oligomeric state changes of Trypanosoma brucei GMP reductase through cystathionine-β-synthase domain
Source: Nat Commun. 2020 Apr 15;11:1837. doi: 10.1038/s41467-020-15611-3 (PMC7160140; doi:10.1038/s41467-020-15611-3)
Supplement: Supplementary file 1 — Supplementary Information [file 41467_2020_15611_MOESM1_ESM.pdf]

Supplementary Information

Allosteric regulation accompanied by oligomeric state changes of *Trypanosoma brucei* GMP reductase through cystathionine-β-synthase domain

Akira Imamura and Tetsuya Okada, *et al.*

This PDF file contains:

- Supplementary Figure 1-13
- Supplementary Table 1-7

Supplementary Figure 1

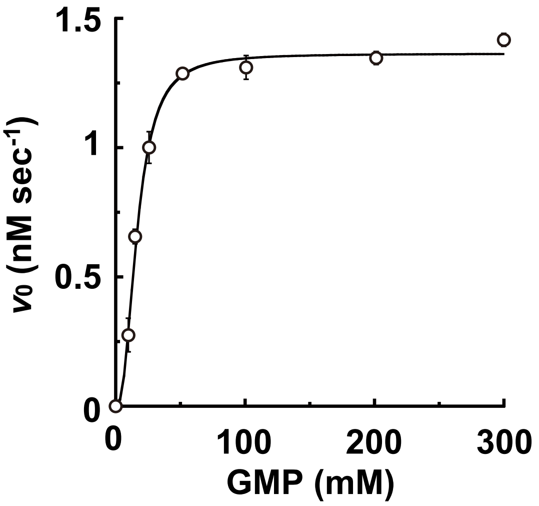

Supplementary Figure 1 | Kinetic analysis of untagged recombinant TbGMPR with varied concentrations of GMP substrate. Recombinant TbGMPR was prepared with pGEX 6P-1 expression and purification system (GE healthcare Japan) as described previously (Bessho, T. *et al.* (2016) *PLoS Negl. Trop. Dis.* **10**, e0004339). The initial velocities at the various concentrations of GMP were determined as described in Methods section, except that the concentrations of TbGMPR and NADPH were 15 µg/mL and 20 µM, respectively, and the reaction temperature was set at 25°C. Note that the data were fitted to the Hill equation, indicating that GMP has a positive cooperativity effect on the enzyme. The V_{max}, $K_{0.5}$ and n_{Hill} constant were calculated as 1.37 ± 0.02 nM·sec^{-1}, 16.4 ± 0.5 µM and 2.38 ± 0.17, respectively. Data were obtained from three independent experiments (n = 3). Each data point represents a mean ± s.d. in error bars. Source data are provided as a Source Data file.

Supplementary Figure 2

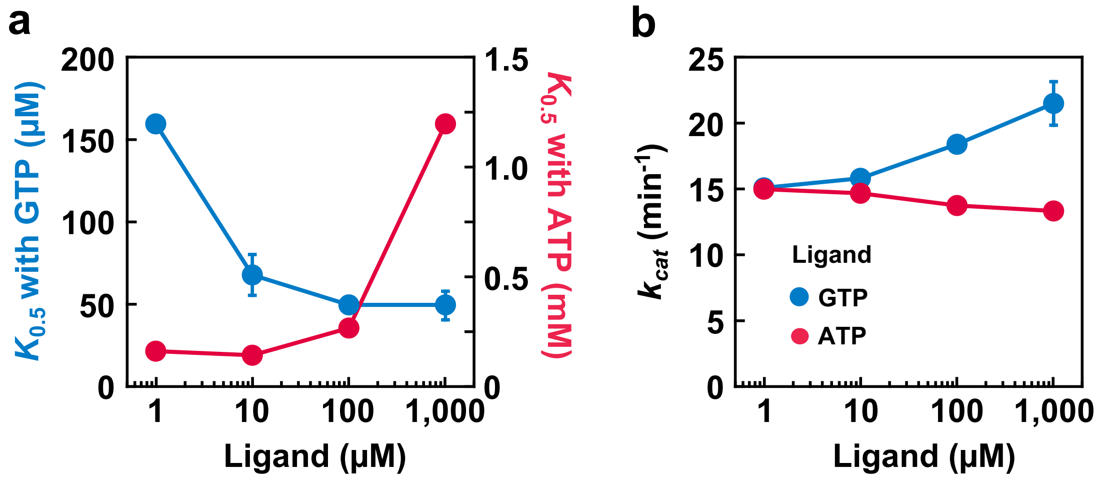

Supplementary Figure 2 | Opposed effects of GTP and ATP as ligands on kinetics of TbGMPR. Changes in $K_{0.5}$ (**a**) and k_{cat} (**b**) in the presence of various concentrations of GTP (blue) or ATP (red). Data were obtained from three independent experiments (n = 3). Each data point represents a mean ± s.d. in error bars. Source data are provided as a Source Data file.

Supplementary Figure 3

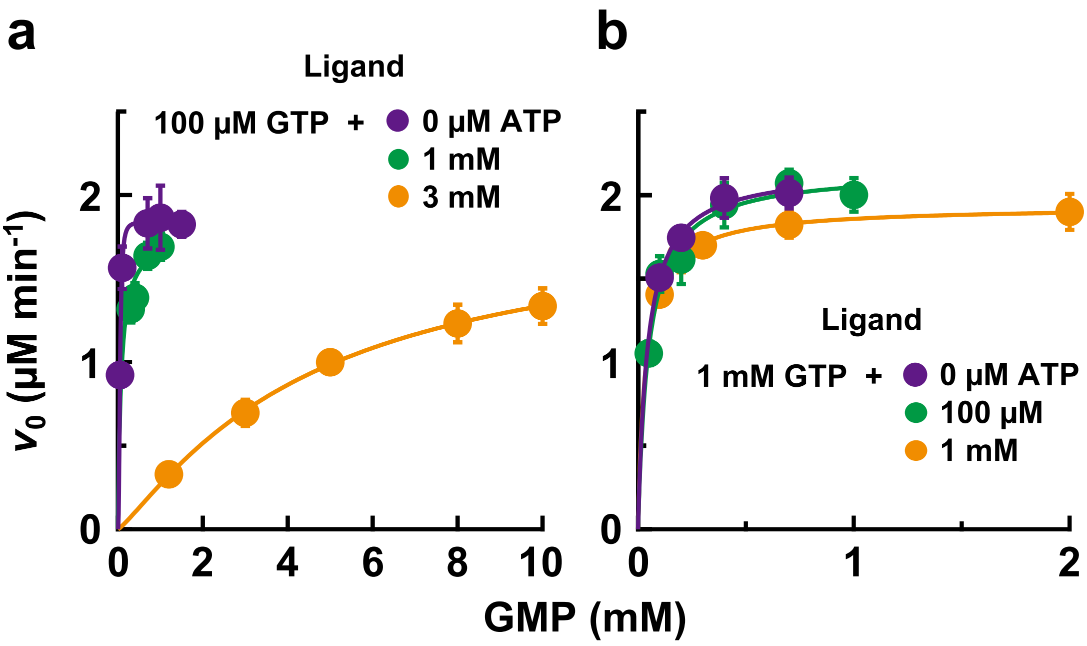

Supplementary Figure 3 | Effect of ATP on TbGMPR activities in the presence of GTP. The initial velocities of TbGMPR activity was monitored in the absence or presence of ATP under fixed concentration of GTP at 100 μM (**a**) or 1 mM (**b**). Data were obtained from three independent experiments (n = 3). Each data point represents a mean ± s.d. in error bars. Source data are provided as a Source Data file.

Supplementary Figure 4

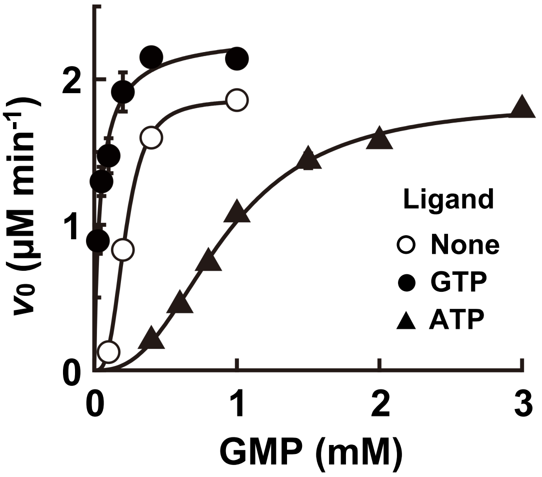

Supplementary Figure 4 | Kinetic analysis of TbGMPR W115R in the presence of purine nucleotides. The initial velocities of TbGMPR W115R were plotted against the concentrations of GMP in the absence (open circles) or presence of 1 mM GTP (closed circles) or ATP (closed triangles) at a fixed concentration of NADPH. Data were fitted to the Hill equation. Data were obtained from three independent experiments (*n* = 3). Each data point represents a mean ± s.d. in error bars. Source data are provided as a Source Data file.

Supplementary Figure 5

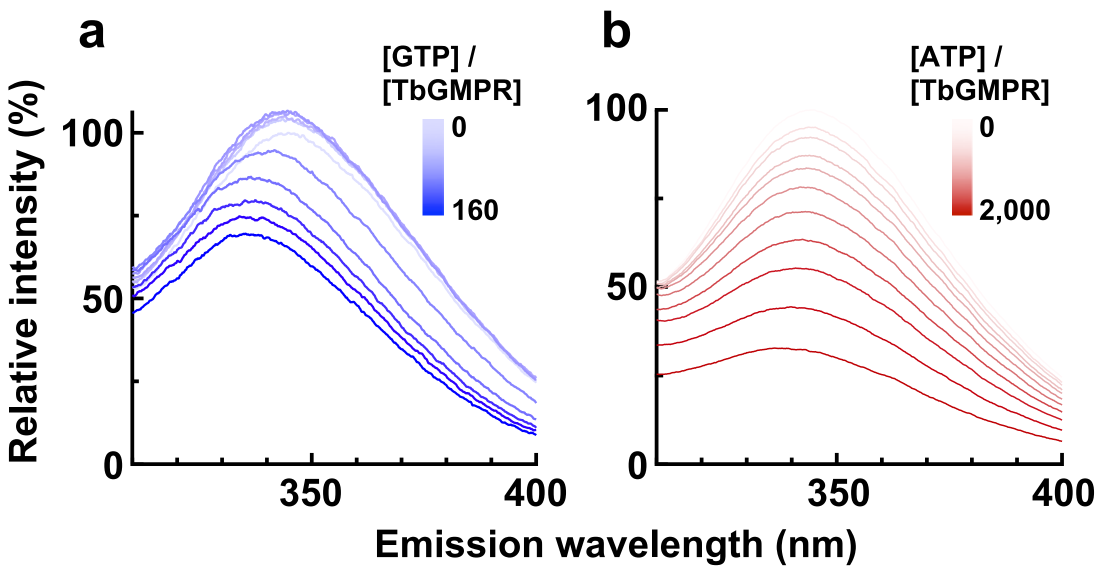

Supplementary Figure 5 | Fluorescence quenching assay of purine nucleotide binding to TbGMPR W115R in the presence of magnesium ion. The fluorescence emission spectra of TbGMPR W115R were measured in EDTA-free buffer containing 1 mM $MgCl_2$. GTP (**a**) and ATP (**b**) were used as ligands. Note that each quenching profile is similar to that observed in the experiment without magnesium ion. Data were obtained from three independent experiments (*n* = 3). Each data point represents a mean ± s.d. in error bars. Source data are provided as a Source Data file.

Supplementary Figure 6

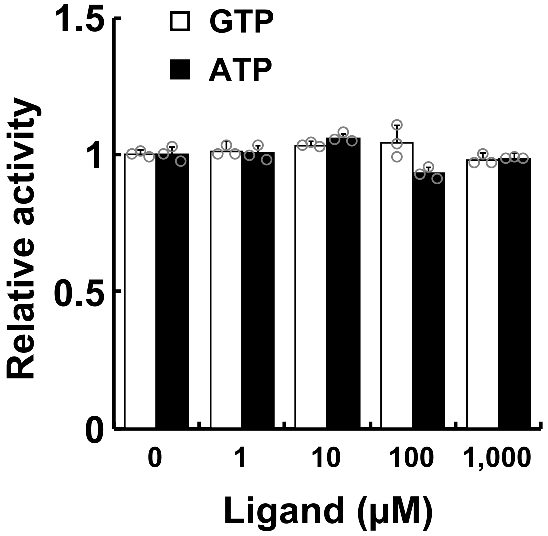

Supplementary Figure 6 | Insensitivity of TbGMPRΔCBS activity to purine nucleotide ligands. The relative activities of TbGMPRΔCBS at the various concentrations of GTP (open) or ATP (filled) in the presence of 1 mM GMP substrate. The activities without the ligands were set to 1. Data were obtained from three independent experiments (*n* = 3). Each data point represents a mean ± s.d. in error bars. Source data are provided as a Source Data file.

Supplementary Figure 7

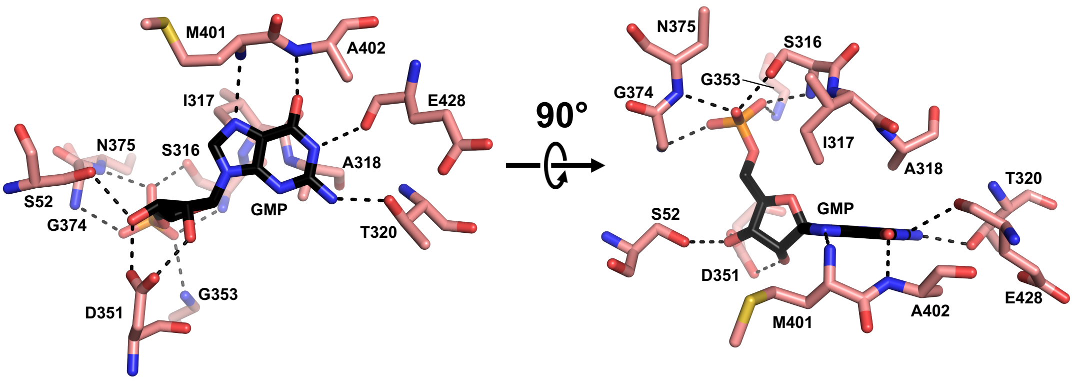

Supplementary Figure 7 | Substrate recognition at the active site in the C318A/GMP complex. The GMP molecule and the amino acid residues interacting with GMP are shown as stick representations. The hydrogen bonds are depicted as dashed lines.

Supplementary Figure 8

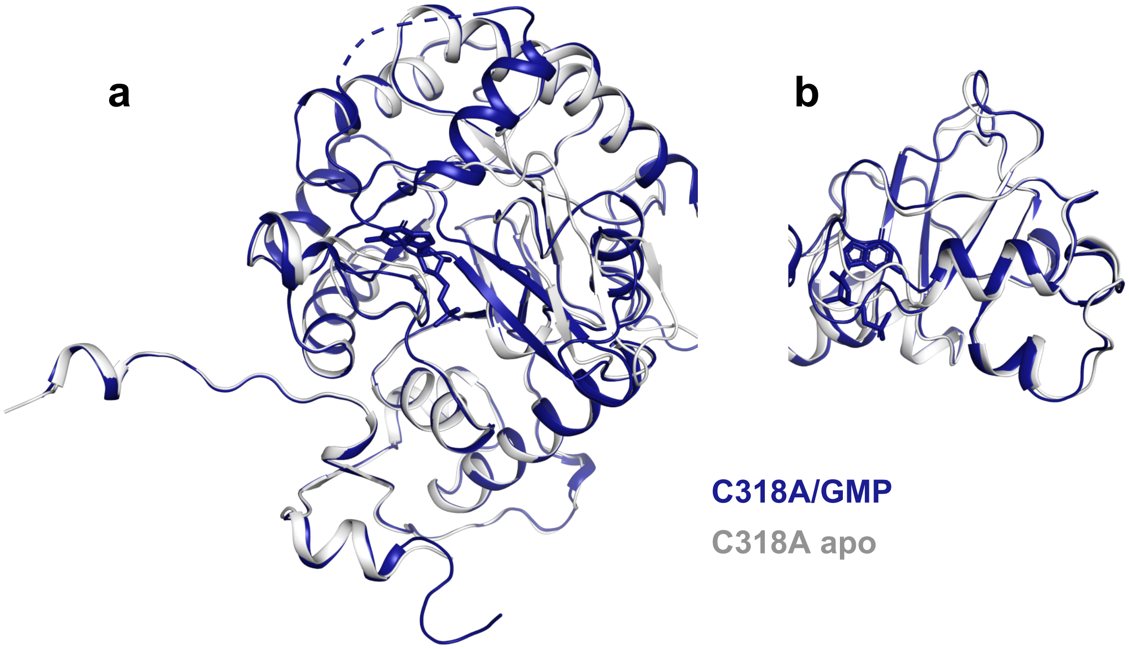

Supplementary Figure 8 | Superposition of C318A/GMP and C318A apo. The structures of C318A/GMP and C318A apo subunits are superimposed with the isolated catalytic (**a**) or CBS (**b**) domain. The structures of C318A/GMP and C318A apo are colored in blue and gray, respectively.

Supplementary Figure 9

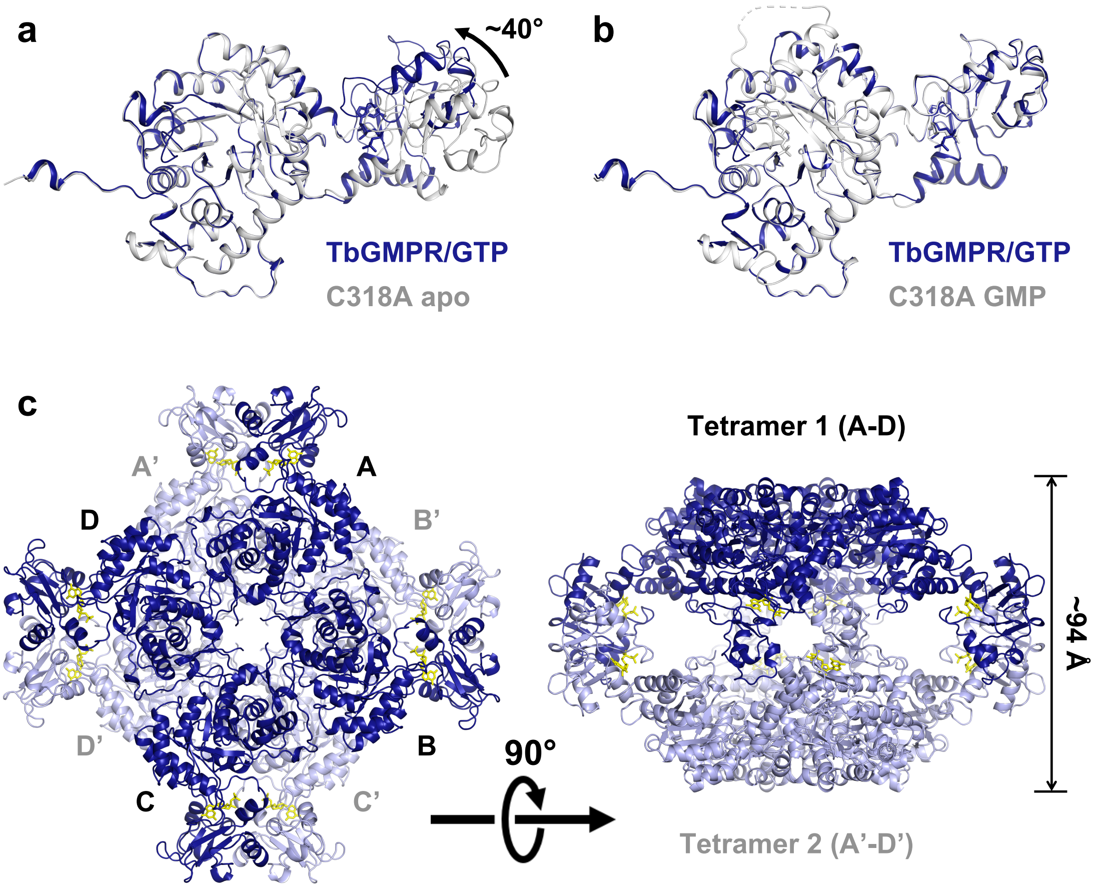

Supplementary Figure 9 | Crystal structure of TbGMPR complexed with GTP. (**a**) The monomeric structures of TbGMPR/GTP (blue) and C318A apo (gray) are represented by a cartoon model with the catalytic domain superimposed. (**b**) The monomeric structure of TbGMPR/GTP (blue) is superimposed in the same way with that of C318A/GMP (gray). (**c**) Cartoon representation of the octameric structure of the TbGMPR/GTP complex, which is composed of two tetramers. Tetramer 1 (subunits A-D) and tetramer 2 (subunits A'-D') are colored in light and dark blue, respectively. GTP molecules also are shown by yellow stick representations.

Supplementary Figure 10

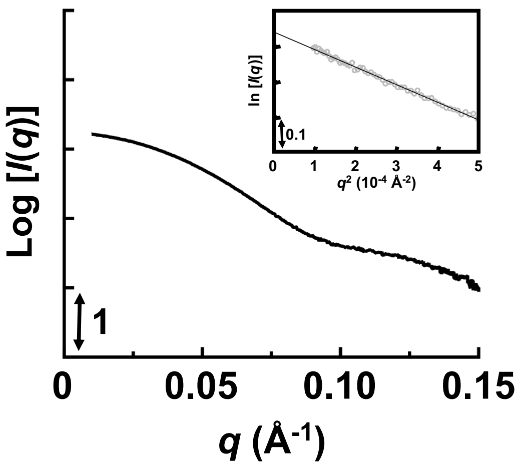

Supplementary Figure 10 | SEC-SAXS analysis of TbGMPRΔCBS. SAXS intensity for TbGMPRΔCBS was plotted against the scattering vector *q*. The inset shows the Guinier plot of the curve. Data were obtained from three independent experiments (*n* = 3). Source data are provided as a Source Data file.

Supplementary Figure 11

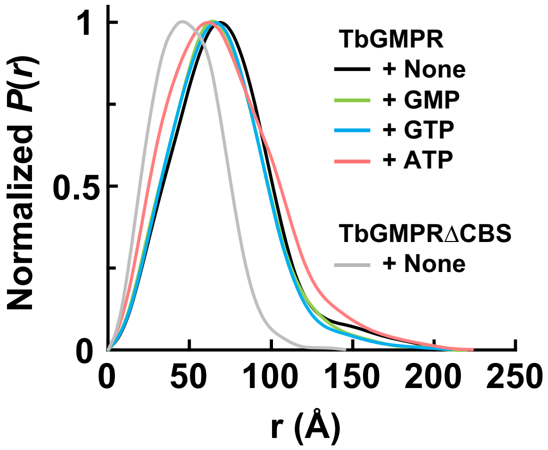

Supplementary Figure 11 | The pair-distance distribution function *P*(*r*) profiles of TbGMPR in the absence or presence of purine nucleotides. The *P*(*r*) profiles were calculated for TbGMPR in the absence (black) or presence of GTP (blue), GMP (green) or ATP (red). The *P*(*r*) profile for TbGMPRΔCBS without ligands is shown in gray. Source data are provided as a Source Data file.

Supplementary Figure 12

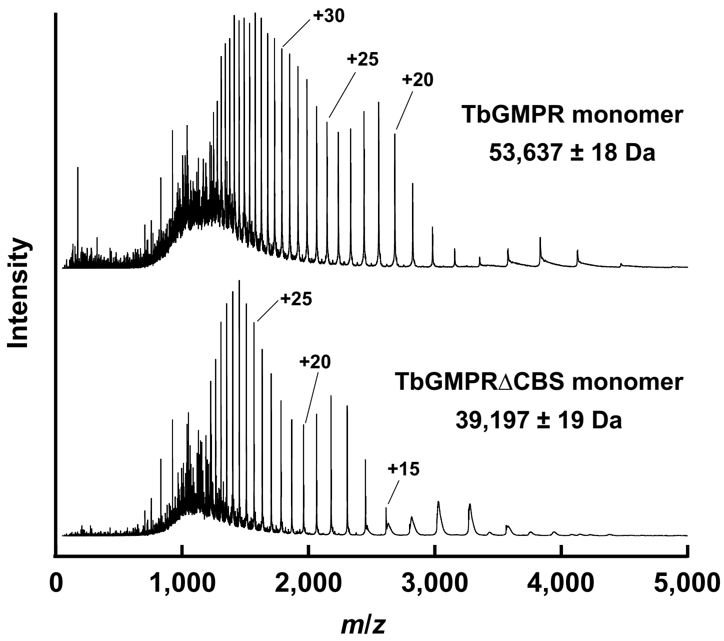

Supplementary Figure 12 | Mass spectra of TbGMPR and TbGMPRΔCBS under denaturing conditions. The mass spectra of TbGMPR and TbGMPRΔCBS under denaturing conditions exhibited ion series with molecular masses of 53,637 ± 18 Da and 39,197 ± 19 Da, respectively. These values are consistent with the respective theoretical masses of 53,627 and 39,184 Da.

Supplementary Figure 13

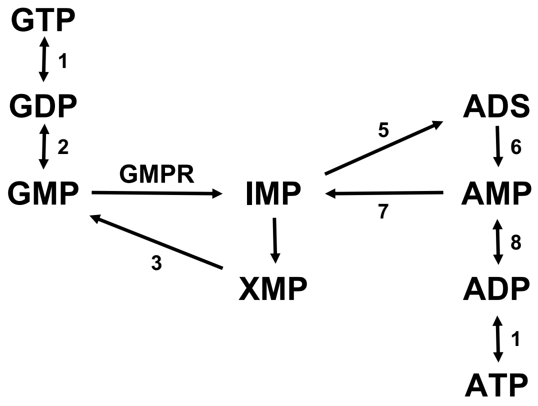

Supplementary Figure 13 | Schematic diagram of salvaging pathways for adenine and guanine nucleotides in *T. brucei*. The enzymes involved in the pathways are designated as numbers: **(1)** Nucleoside-diphosphate kinase, **(2)** guanylate kinase, **(3)** GMP synthetase, **(4)** IMP dehydrogenase, **(5)** adenylosuccinate synthase, **(6)** adenylosuccinate lyase, **(7)** AMP deaminase, **(8)** adenylate kinase. The metabolites shown here are abbreviated as follows: GDP, guanosine 5'-diphosphate; XMP, xanthosine 5'-monophosphate; AMP, adenosine 5'-monophosphate; ADP, adenosine 5'-diphosphate; ADS, adenylosuccinate.

Supplementary Table 1 | Data collection and refinement statistics for TbGMPR structure determination

Crystals<br>PDB ID	C318A apo<br>6JL8	C318A/GMP<br>6JIG	TbGMPR/GTP<br>6LK4
**Data collection**			
**Wavelength (Å)**	1.0	1.0	1.0
**Space group**	*I*422	*I*422	*I*422
**Unit cell dimensions**			
a, b, c **(Å)**	124.56, 124.56, 540.17	143.28, 143.28, 132.96	144.11, 144.11, 135.70
α, β, γ **(deg)**	90, 90, 90	90, 90, 90	90, 90, 90
**Resolution range (Å)[a]**	49.51 - 2.80 (2.97 - 2.80)	48.73 - 1.90 (2.02 - 1.90)	49.40 - 2.50 (2.65 - 2.50)
**Total reflections**	681439 (111274)	1381741 (206368)	606019 (71062)
**Unique reflections**	52835 (8338)	54159 (8636)	24874 (3926)
$I/\sigma(I)$ [a]	18.2 (3.3)	53.6 (3.6)	43.4 (3.2)
**Redundancy [a]**	12.9 (13.3)	25.5 (23.9)	24.4 (18.1)
**Completeness (%)[a]**	99.9 (99.4)	99.9 (99.5)	99.9 (99.5)
R_{merge} **(%)[a,b]**	14.1 (91.0)	3.9 (98.8)	6.5 (92.1)
$CC_{1/2}$ [a]	99.9 (92.7)	100.0 (91.0)	100.0 (90.6)
**Wilson** ***B*****-factor** ($Å^2$)	50.5	38.2	57.8
**No. of molecules per asymmetric unit**	2	1	1
**Refinement**			
**Resolution range (Å) [a]**	21.81 - 2.80 (2.90 - 2.80)	27.35 - 1.90 (1.97 - 1.90)	33.92 - 2.50 (2.59 - 2.50)
**No. of atoms per asymmetric unit**			
**Protein**	6115	3357	2980
**Ligand/ion**	—	49	33
**Solvent**	19	95	3
**R.m.s deviation from ideal values**			
**Bond lengths (Å)**	0.009	0.007	0.008
**Bond angles (deg)**	1.05	0.88	0.96
R_{work} **(%)[a,c]**	22.3 (30.8)	20.4 (27.2)	20.6 (31.9)
R_{free} **(%)[a,d]**	25.8 (35.9)	22.4 (30.5)	24.4 (37.9)
**Ramachandran plot statistics (%)**			
**Residues in favoured regions**	92.0	97.5	95.7
**Residues in allowed regions**	7.2	2.5	4.3
**Outliers (%)**	0.8	0.0	0.0
B**-factor** ($Å^2$)			
**Protein**	66.8	46.1	65.0
**Ligand/ion**	—	44.9	71.0
**Solvent**	45.8	43.6	58.5

[a] Values in parentheses are for the highest resolution shell. [b] $R_{merge} = \Sigma_h \Sigma_i |I_{h,i} - \langle I_h \rangle| / \Sigma_h \Sigma_i I_{h,i}$, where $I_{h,i}$ is the intensity value of the ith measurement of h and $\langle I_h \rangle$ is the corresponding mean value

of I_h for all i measurements. [c] $R_{work} = \Sigma||F_o| - |F_c|| / \Sigma |F_o|$. [d] R_{free} is an R factor of the refinement evaluated for 5% of reflections that were excluded from the refinement.

Supplementary Table 2 | Intermolecular interactions between adjacent subunits in each tetramer

Hydrogen bond interactions

Atoms in subunit A[#]		Atoms in subunit B[#]		Distance (Å)			
				C318A apo		C318A/GMP	TbGMPR/GTP
Residue	Atom	Residue	Atom	Tetramer 1[*]	Tetramer 2[*]		
Ile 20	N	Gly 325	O	2.7	2.9	2.8	2.7
Ile 20	O	Gly 327	N	2.8	2.9	2.9	3.0
Gln 23	O	His 262	NE2	2.8	2.7	2.8	3.0
His 24	NE2	Asp 266	OD1		3.2		
Ser 25	N	His 264	O	3.1	3.1	3.0	3.0
Ser 25	O	Asp 266	N	3.1	3.1	3.1	3.0
Ser 332	OG	Ser 6	OG	2.9		2.8	3.0
Thr 356	OG1	Leu 322	O			3.0	3.3
Ser 357	OG	Val 323	O	2.6	2.4	2.6	2.5
Gly 358	N	Leu 322	O	2.9	2.9	3.0	3.0
Ser 361	OG	Ala 324	O	3.1	3.3	3.0	3.1
Ser 453	OG	Glu 428	OE2			2.6	
Tyr 457	OH	Ala 261	O	3.1			
Arg 474	N	Pro 10	O	2.8	2.7	2.7	2.8
Arg 474	O	Gly 12	N	3.0	2.7	2.7	2.8
Thr 476	OG1	Asp 17	OD1	2.6	2.7	2.5	2.5
Gly 479	O	Arg 321	NH1	2.6	3.1		
Gly 479	O	Arg 321	NH2	3.2			
Leu 480	O	Arg 321	NH2		2.7		
Glu 482	OE1	Arg 321	NH2	2.6			
Glu 482	OE2	Thr 14	OG1	2.4			
Ser 483	OG	Arg 321	NH1			3.1	
His 486	N	Ser 316	O			3.1	
His 486	NE2	Pro 314	O			2.9	
Gly 487	O	Asn 375	ND2			3.3	

Hydrophobic interactions

Atoms in subunit A[#]		Atoms in subunit B[#]		Distance (Å)			
				C318A apo		C318A/GMP	TbGMPR/GTP
Residue	Atom	Residue	Atom	Tetramer 1[*]	Tetramer 2[*]		
Pro 10	CG	Phe 3	CD2				3.7
Leu 13	CD1	Ser 6	CB				3.9
Leu 13	CD1	Ile 9	CD1	3.8			3.8
Leu 19	CD1	Arg 321	CD	3.9		3.9	
Leu 19	CD1	Arg 321	CZ				3.7
Leu 19	CD1	Gly 327	CA				3.8
Leu 19	CD1	Gly 327	C		3.8	3.6	3.5
Leu 19	CD1	Pro 329	CD		3.7		
Leu 19	CD2	Arg 321	CB			3.9	

Leu 19	CD2	Arg 321	CD	3.4			3.8
Ile 20	CB	Ala 326	CA	3.8	3.9		
Ile 20	CD1	Gly 325	C	3.9			
Pro 22	CB	His 262	CE1	3.5	3.5	3.7	3.8
Pro 22	CG	Val 312	CB		3.8		
Pro 22	CG	Val 312	CG1	3.6			3.6
Pro 22	CD	Val 328	CG2	3.8	3.7	3.7	3.8
His 24	CA	His 264	CG		3.8	3.9	3.9
His 24	CA	His 264	CD2		3.9		
His 24	CA	His 264	CE1	3.9		3.7	3.8
His 24	CB	His 264	CD2	3.9	3.8		
His 24	CB	His 264	CE1	3.6	3.8	3.6	3.7
His 24	CE1	Asp 266	CG	3.6		3.7	3.8
Ser 25	CB	His 262	CD2	3.5		3.7	3.9
Val 328	CG1	Phe 3	CE1	3.5		3.7	
Val 328	CG1	Phe 3	CZ		3.7	3.5	
Ser 332	CB	Phe 3	CD1				3.7
Ser 332	CB	Phe 3	CE1				3.5
Ser 332	CB	Ser 6	CB		3.7		
Leu 335	CD1	Glu 5	C		3.8		
Leu 335	CD1	Glu 5	CB		3.9		
Gly 358	CA	Val 323	C		3.7	3.8	3.7
Gly 358	CA	Ala 324	C	3.6	3.7	3.7	3.7
Gly 358	CA	Gly 325	CA	3.8		3.8	3.7
Lys 362	CE	Gly 325	CA	3.7			3.8
Gly 450	CA	Val 323	CG1	3.8	3.9		3.7
Ser 453	C	Ala 324	CB			3.9	
Ser 453	CB	Ala 324	CB			3.8	
Gly 454	CA	Ala 324	CB		3.7	3.8	3.8
Tyr 457	CA	His 262	CD2			3.9	
Tyr 457	CB	Ala 326	CB	3.6	3.6	3.6	3.9
Tyr 457	CG	Ala 326	CB	3.4	3.4	3.5	3.7
Tyr 457	CD1	His 262	CG	3.7	3.7	3.7	3.8
Tyr 457	CD1	Ala 326	CB	3.9	3.9		
Tyr 457	CD2	Ala 324	CB	3.8	3.6	3.8	3.8
Tyr 457	CD2	Ala 326	CB	3.7	3.6	3.8	
Tyr 457	CE1	His 262	CB	3.6	3.6	3.6	3.7
Tyr 457	CE1	His 262	CG	3.7	3.8	3.7	3.7
Tyr 457	CE1	Val 312	CG1	3.8		3.8	3.7
Tyr 457	CE2	Thr 320	CG2		3.7	3.9	
Tyr 457	CE2	Ala 324	CB	3.9	3.7		
Tyr 457	CZ	Thr 320	CG2	3.9	3.6	3.8	3.8
Phe 472	C	Ile 9	CG2	3.9	3.4	3.7	3.9
Phe 472	CB	Ile 9	CG2		3.8		
Val 473	CA	Ile 9	CG2	3.8	3.6	3.9	
Val 473	C	Ile 9	CG2	3.8	3.8		

Val 473	CG2	Gly 12	CA	3.9		3.8
Arg 474	CB	Thr 11	CA	3.9	3.8	
Arg 474	CG	Ile 9	CG2	3.9		
Met 475	CG	Pro 329	CG		3.8	
Ala 478	C	Thr 14	CG2			3.7
Ala 478	CB	Thr 14	CG2			3.9
Ala 478	CB	Asp 16	CB		3.9	3.7
Ala 478	CB	Asp 17	CG			3.9
Gly 479	CA	Thr 14	CG2	3.8		
Glu 482	C	Arg 321	CZ		3.5	
Glu 482	CG	Pro 314	CA	3.8		
Glu 482	CG	Pro 314	CB	3.9		
Glu 482	CD	Pro 314	CA	3.9		
Glu 482	CD	Pro 314	CB	3.4		
Ser 483	CA	Arg 321	CD			3.6
Ser 483	CB	Arg 321	CB	3.6		
Ser 483	CB	Arg 321	CG	3.6		
Ser 483	CB	Arg 321	CD	3.5		3.5
Ser 483	CB	Arg 321	CZ	3.8		
Gly 484	CA	Leu 322	CD1			3.9
His 486	CE1	Ser 316	CA			3.5
Ala 489	C	Gly 432	CA			3.7

[#] The interactions between subunits B-C, C-D, and D-A were identical to those between subunits A-B.

[*] The intermolecular interactions were analyzed individually for each tetramer (Tetramer 1 or 2).

Supplementary Table 3 | Intermolecular interactions between the tetramers

Hydrogen bond interactions

Atoms in subunit A		Atoms in subunit A'		Distance (Å)		
Residue	Atom	Residue	Atom	C318A apo	C318A/GMP	TbGMPR/GTP
Leu152	O	Arg187	NH1		2.4	2.5
Met163	O	Arg190	NH2			2.9
Arg187	NH1	Leu152	O	2.9	2.4	2.5
Arg190	NH2	Met163	O			2.9

Hydrophobic interactions

Atoms in subunit A		Atoms in subunit A'		Distance (Å)		
Residue	Atom	Residue	Atom	C318A apo	C318A/GMP	TbGMPR/GTP
His148	CD2	Leu209	CD2	3.5	3.6	
Lys151	CD	Leu209	CD2	3.7	3.8	
Leu152	CB	Arg187	CG		3.6	3.8
Leu152	CG	Leu209	CD1	3.8	3.7	3.6
Leu152	CD1	Met186	CB		3.7	3.7
Leu152	CD1	Arg187	CG		3.8	
Leu152	CD1	Leu209	CD1	3.6	3.9	3.5
Leu152	CD2	Thr183	CG2	3.7		
Leu152	CD2	Leu209	CD1		3.7	3.7
Leu152	CD2	Arg216	CD	3.9		
Leu162	C	Arg190	CZ			3.8
Met186	CB	Leu152	CD1		3.7	3.7
Arg187	CG	Leu152	CB		3.6	3.8
Arg187	CG	Leu152	CD1	3.5	3.8	
Arg190	C	His148	CE1	3.7		
Arg190	CZ	Leu162	C			3.8
Leu209	CD1	Leu152	CG	3.7	3.7	3.6
Leu209	CD1	Leu152	CD1	3.9	3.9	3.5
Leu209	CD1	Leu152	CD2	3.6	3.7	3.7
Leu209	CD2	His148	CD2	3.7	3.6	
Leu209	CD2	Lys151	CD	3.9	3.8	
Arg216	CD	Leu152	CD2	3.6		

The interactions between subunits B-B', C-C', and D-D' were identical to those between subunits A-A'.

Supplementary Table 4 | Interactions between substrate and amino acid residues at the active site in C318A/GMP

Hydrogen bond interactions

GMP atom	Residue	Atom	Distance (Å)
N1	Glu428	O	3.0
N2	Thr320	OG1	2.5
O6	Ala402	N	2.7
N7	Met401	N	3.1
O2'	Asp351	OD2	2.4
O3'	Ser52	OG	2.8
O3'	Asp351	OD1	2.4
O1P	Ser316	OG	2.7
O1P	Asn375	N	2.8
O2P	Ser316	N	2.7
O2P	Gly353	N	3.0
O3P	Gly374	N	2.8

Hydrophobic interactions

GMP atom	Residue	Atom	Distance (Å)
C2	Ala318	CA	3.9
C2	Ala318	CB	3.2
C4	Ala318	CB	3.9
C5	Ile317	CD1	3.3
C6	Ile317	CD1	3.9
C6	Ala402	CB	3.8
C8	Ile317	CD1	3.4

Supplementary Table 5 | Interactions between ligands and amino acid residues at the allosteric regulatory site of C318A/GMP and TbGMPR/GTP

Hydrogen bond interactions

			Distance (Å)	
**GMP/GTP atom**	**Residue**	**Atom**	**C318A/GMP**	**TbGMPR/GTP**
N2	Cys129	N		3.1
N2	Cys129	O	3.3	
N2	Cys129	SG	3.3	
O6	Arg93	NH2	3.1	3.1
O6	Arg103	N	2.9	3.0
N7	Arg93	NE	3.1	3.1
O2'	Asp211	OD2	2.5	2.5
O3'	Ala94	O	3.1	
O3'	Asp211	OD1	2.7	3.0
O3'	Lys214	NZ		3.3
O1P	Ser210	OG	2.8	
O2P	Gly128	N	3.0	

Hydrophobic interactions

			Distance (Å)	
**GMP/GTP atom**	**Residue**	**Atom**	**C318A/GMP**	**TbGMPR/GTP**
C2	Val127	CB	3.7	3.6
C2	Leu206	CD2		3.8
C4	Ile99	CD1	3.8	3.9
C4	Val127	CB		3.8
C4	Val127	CG1	3.7	3.7
C5	Ile99	CG2	3.8	
C5	Val127	CB		3.8
C5	Val127	CG1	3.5	3.4
C6	Val127	CB		3.7
C6	Val127	CG1	3.6	3.6
C8	Arg93	CG	3.8	
C8	Arg123	CD	3.7	3.7
C1'	Arg123	CD	3.6	3.5
C1'	Arg123	CZ		3.7
C2'	Ile99	CD1	3.7	3.8
C2'	Asp211	CG	3.9	
C3'	Asp211	CG	3.7	
C4'	Arg123	CZ	3.6	3.4
C5'	Gly128	CA		3.8

Supplementary Table 6 | Structural parameters of TbGMPR observed in SEC-SAXS analysis

**Data-collection parameter**					
Instrument	BioSAXS beamline B21 (Diamond Light Source)				
Optics	Double multilayer monochromator, focusing bend toroidal mirror				
Wavelength (Å)	1.0				
q range	0.003-0.35				
Exposure time (sec)	3				
Temperature (K)	293				
**Software employed**					
Primary data reduction	DAWN processing pipeline				
Data processing and analysis	ScÅtter				
Calculation of oligomeric state	OLIGOMER				
Computation of model intensities	FFMAKER				
**Structural parameters and Molecular-mass determination†**					
**TbGMPR**	**WT**	**WT**	**WT**	**WT**	**ΔCBS**
**Ligand**	**None**	**GMP**	**GTP**	**ATP**	**None**
$I(0)$ (cm^{-1}) [from $P(r)$]	0.256	0.244	0.268	0.168	0.176
R_g (Å) [from $P(r)$]	55.4 ± 2.59	53.2 ± 3.32	52.4 ± 2.12	55.7 ± 3.48	37.8 ± 0.72
$I(0)$ (cm^{-1}) (from Guinier)	0.253	0.239	0.263	0.165	0.176
R_g (Å) (from Guinier)	55.4 ± 0.56	52.9 ± 0.56	52.8 ± 0.41	55.3 ± 0.68	38.2 ± 0.18
D_{max} (Å)	210.0	209.5	210.5	223.5	145.0
Porod volume estimate (x 10^5 Å^3)	9.58	8.56	8.10	7.81	3.01
Monomeric dry volume calculated from sequence (x 10^5 Å^3)	0.65	0.65	0.65	0.65	0.48
Molecular mass M_r from volume of correlation (kDa)	494	453	462	314	137
Calculated monomeric M_r from sequence (kDa)	53.8	53.8	53.8	53.8	39.3

† The molecular mass was derived from the volume of correlation which was directly estimated from the subtracted 1-D scattering curve (Rambo, R.P. and Tainer, J.A. (2013) *Nature*. **496**, 477–481).

Supplementary Table 7 | Primers used in this study

**Cloning**	
Forward	5’-GGGAATTCCATATGTCCTTCAATGAATCGGCATC-3’
Reverse	5’-CCCAAGCTTAAGTTTGGCAACACCGTGAC-3’
**ΔCBS mutation**	
Forward	5’-GGTCGCCTCCTTGTCGG-3’
Reverse	5’-GGACTGTGCGCGCTTC-3’
**W115R mutation**	
Forward	5’-CGGGAGGGGTTGAACTGGAA-3’
Reverse	5’-TGCCTCACGAGCCGTTTCG-3’
**C318A mutation**	
Forward	5’-GCCATTACCCGCCTCGTTG-3’
Reverse	5’-AATACTCCCAGGGCCGACAC-3’
