# Supplementary material for: Allosteric regulation accompanied by oligomeric state changes of Trypanosoma brucei GMP reductase through cystathionine-β-synthase domain
Source: Nat Commun. 2020 Apr 15;11:1837. doi: 10.1038/s41467-020-15611-3 (PMC7160140; doi:10.1038/s41467-020-15611-3)
Supplement: Supplementary file 3 — Reporting Summary [file 41467_2020_15611_MOESM3_ESM.pdf]

nature research

Corresponding author(s): Takashi Inui

Last updated by author(s): Mar 17, 2020

Reporting Summary

Nature Research wishes to improve the reproducibility of the work that we publish. This form provides structure for consistency and transparency in reporting. For further information on Nature Research policies, see Authors & Referees and the Editorial Policy Checklist .

Statistics

For all statistical analyses, confirm that the following items are present in the figure legend, table legend, main text, or Methods section.

n/a	Confirmed	
☐	☒	The exact sample size (n) for each experimental group/condition, given as a discrete number and unit of measurement
☐	☒	A statement on whether measurements were taken from distinct samples or whether the same sample was measured repeatedly
☒	☐	The statistical test(s) used AND whether they are one- or two-sided<br>*Only common tests should be described solely by name; describe more complex techniques in the Methods section.*
☒	☐	A description of all covariates tested
☒	☐	A description of any assumptions or corrections, such as tests of normality and adjustment for multiple comparisons
☐	☒	A full description of the statistical parameters including central tendency (e.g. means) or other basic estimates (e.g. regression coefficient) AND variation (e.g. standard deviation) or associated estimates of uncertainty (e.g. confidence intervals)
☒	☐	For null hypothesis testing, the test statistic (e.g. F, t, r) with confidence intervals, effect sizes, degrees of freedom and P value noted<br>*Give P values as exact values whenever suitable.*
☒	☐	For Bayesian analysis, information on the choice of priors and Markov chain Monte Carlo settings
☒	☐	For hierarchical and complex designs, identification of the appropriate level for tests and full reporting of outcomes
☒	☐	Estimates of effect sizes (e.g. Cohen's d, Pearson's r), indicating how they were calculated

Our web collection on statistics for biologists contains articles on many of the points above.

Software and code

Policy information about availability of computer code

Data collection	The absorption was monitored by UV-2600 spectrophotometer (Shimadzu) or Eon microplate reader (BioTek, VT) and the associated commercial software. Fluorescence quenching data were collected with F-7000 Fluorescence Spectrophotometer (Hitachi High-Technologies). X-ray diffraction data were collected with 1.0-Å synchrotron radiation on beamline BL38B1 at SPring-8 (Harima, Japan). The SEC-SAXS experiments were performed at beamline B21, Diamond Light Source (Didcot, UK).
Data analysis	Data plot and curve fitting analysis was performed by IGOR Pro 6.3(WaveMetrics, OR).<br>X-ray diffraction data were indexed, merged and scaled using the XDS program package (ref. 24). Structures were determined by molecular replacement with Phaser and Molrep (ref. 26, 27). Iterative rounds of model building and refinement were carried out by a combination of COOT, PHENIX, and Refmac5 in the CCP4i software package (ref. 28-31). The structure and the crystal packing were analyzed with PyMOL (http://www.pymol.org) and PISA (ref. 13), respectively.<br>Raw SAXS 2-D images were processed with the software package DAWN (ref. 32; https://dawnsci.org) to produces 1-D SAXS curves, and the curves were further analyzed using ScÅtter (ref. 33; http://www.bioisis.net). The oligomeric states of TbGMPR were calculated by OLIGOMER with fitting to the form-factor files calculated by FFMAKER based on the crystal structures of TbGMPR (ref. 35).

For manuscripts utilizing custom algorithms or software that are central to the research but not yet described in published literature, software must be made available to editors/reviewers. We strongly encourage code deposition in a community repository (e.g. GitHub). See the Nature Research guidelines for submitting code & software for further information.

Data

Policy information about availability of data

All manuscripts must include a data availability statement. This statement should provide the following information, where applicable:

- Accession codes, unique identifiers, or web links for publicly available datasets
- A list of figures that have associated raw data
- A description of any restrictions on data availability

Coordinates and structure factors have been deposited in the Protein Data Bank under accession number 6JL8, 6JIG, and 6IHY. Other data are available from corresponding author upon reasonable request.

Field-specific reporting

Please select the one below that is the best fit for your research. If you are not sure, read the appropriate sections before making your selection.

- [x] Life sciences
- [ ] Behavioural & social sciences
- [ ] Ecological, evolutionary & environmental sciences

For a reference copy of the document with all sections, see nature.com/documents/nr-reporting-summary-flat.pdf

Life sciences study design

All studies must disclose on these points even when the disclosure is negative.

Sample size	Sample size was set to minimum numbers in order to analyze the differences between the experimental data. This was also based on the sample size used in the studies accepted from the peer-reviewed journals (Bessho T. et al. Parasitology. 2013. 140(6):735-45; Nakatsuji M. et al. PLoS One. 2015. Nov 3;10(11):e0142206).
Data exclusions	No samples or data were excluded.
Replication	Kinetic analysis, fluorescence quenching assay and SEC-SAXS data were obtained from three independent experiments (N=3).
Randomization	No randomization was performed.
Blinding	No blinding was performed.

Reporting for specific materials, systems and methods

We require information from authors about some types of materials, experimental systems and methods used in many studies. Here, indicate whether each material, system or method listed is relevant to your study. If you are not sure if a list item applies to your research, read the appropriate section before selecting a response.

Materials & experimental systems

n/a	Involved in the study	
[x]	[ ]	Antibodies
[x]	[ ]	Eukaryotic cell lines
[x]	[ ]	Palaeontology
[x]	[ ]	Animals and other organisms
[x]	[ ]	Human research participants
[x]	[ ]	Clinical data

Methods

n/a	Involved in the study	
[x]	[ ]	ChIP-seq
[x]	[ ]	Flow cytometry
[x]	[ ]	MRI-based neuroimaging
